# Uncovering Cognitive Subtypes in Essential Tremor: A Data-Driven Clustering Approach in Cognitively Normal Individuals

**DOI:** 10.5334/tohm.1090

**Published:** 2025-11-05

**Authors:** Alessia Sarica, Camilla Calomino, Rita Nisticò, Maria Salsone, Andrea Quattrone, Aldo Quattrone, Fabiana Novellino

**Affiliations:** 1 Neuroscience Research Center, University “Magna Graecia”, Catanzaro, Italy; 2 Department of Medical and Surgical Sciences, University “Magna Graecia”, Catanzaro, Italy; 3 Vita-Salute San Raffaele University, 20132 Milan, Italy; 4 IRCCS Policlinico San Donato, San Donato Milanese, 20097 Italy; 5 Institute of Neurology, Department of Medical and Surgical Sciences, Magna Graecia University, Catanzaro, Italy

**Keywords:** essential tremor, memory, cluster analysis, normal cognition

## Abstract

**Background:**

Different profiles of cognitive functioning have been demonstrated in ET subjects, also in patients with normal cognition. However, the prognostic significance of these profiles remains still debated. In this study, we aimed to explore different cognitive patterns among cognitively normal ET subjects and their relationship with the cognitive profiles of healthy subjects.

**Methods:**

We enrolled 50 cognitively normal subjects (26 ET and 24 age-, sex-, and education-matched healthy subjects), which scored within normal ranges individually in all tests of a comprehensive neuropsychological battery covering memory, executive function, attention, visuospatial abilities, and language. Unsupervised clustering was applied separately within each group. Cluster membership was validated by post-hoc comparisons using ANOVA and Bonferroni-corrected pairwise tests to compare the variables among the clusters.

**Results:**

All HC clustered together into a single high-functioning cognitive profile. On the contrary, we found two different clusters within ET, C1 (n = 14), showing high performance across all domains, and C2 (n = 12) which exhibited significantly poorer performances in the RAVLT-IR (p < 0.0001), RAVLT-DR (p = 0.0002), and Digit Span Forward (p = 0.015) than both ET-C1 and HC subjects. Other domains showed no significant differences across ET clusters.

**Discussion:**

This study demonstrates a cognitive heterogeneity in ET and reveals a memory-impaired subgroup absent among HC. The ET cluster with lower memory performance likely reflects a pattern of vulnerability for longitudinal decline or progression to mild cognitive impairment. The identification of this profile has relevant translational implications for prognosis and identification of early intervention strategies.

**Highlights:**

A data-driven clustering approach was applied to cognitive variables in HC subjects and ET patients. HC formed a homogeneous cluster. ET were divided into two cognitive subgroups: one cluster with high performance, and one memory-impaired cluster, significantly diverging from both the intact ET subgroup and HC. This may represent a cognitively vulnerable ET subgroup, with strong implications for targeted screening, early neuroprotective interventions and personalized clinical management.

## Introduction

Essential tremor (ET) is the most encountered movement disorder [1]. It is a chronic pathology with a very slow progression, characterized by the appearance of tremor, which constitutes the predominant disorder of this disease [2].

For many years, it was believed to be a pathology with an overall “benign” prognosis, especially when compared to other diseases that produce tremor and are much more aggressive. However, in recent years, this consideration has been largely revisited [3]. It is now clear that ET is a pathology that can be disabling, both due to the effects of tremor itself, inducing embarrassment and often significant incapacitation, and because other non-motor symptoms can occur, which can make the pathology more crippling, frequently necessitating lifestyle adjustments for affected individuals [4,5].

While non-motor symptoms have traditionally been underrecognized, cognitive disorders have focused some attention and debate in recent years. Epidemiological studies have demonstrated a higher risk of cognitive decline in ET population compared to age- and education-matched healthy controls [6,7]. In ET patients, the annual conversion rates from normal cognition to MCI and from MCI to dementia are approximately three times that of the general population [8]. Attention, executive functions, memory and visuospatial skills are the most affected domains [9].

Despite growing recognition of cognitive disorders in ET, little is known about the inter-individual variability in cognitive functioning among patients who are considered cognitively normal based on standard clinical assessments. Emerging evidence suggests that even within this apparently homogeneous group, distinct cognitive patterns may exist. Previous research has identified distinct clusters of cognitive profiles in ET subjects whose functions fall within the normal range [10]: one cluster with high cognitive performance, coexisting with two others with slight but measurable declines in memory and attentional/visuospatial functions, respectively. These findings are interesting in the debate about cognitive functioning in ET. Indeed, it remains still not clarified whether this variability within apparently normal cognitive profiles should be interpreted as subclinical onset of a deterioration in cognitive functions or simply reflect a physiological variability. However, this evidence suggests the hypothesis that subjects with initial cognitive vulnerability may not be identified through traditional diagnostic methods.

Conventional neuropsychological tests remain the gold standard for evaluating cognitive performance and their diagnostic use typically relies on predefined cut-offs that classify individuals as either cognitively normal or impaired. This binary approach may overlook subtle inter-individual variability within the normal range. To better capture this hidden heterogeneity, cluster analysis, an unsupervised machine learning approach, has become increasingly valuable because it allows to identify naturally emerging subtypes based solely on patterns in the data [11]. Among available methods, Affinity Propagation (AP) represents a particularly effective and flexible clustering algorithm. Unlike k-means or hierarchical clustering, AP does not require predefining the number of clusters. Instead, it simultaneously estimates both the number and composition of clusters by iteratively exchanging messages between data points and identifying representative exemplars [11]. These characteristics make AP particularly suitable for identify nuanced differences in a heterogeneous population [12] or small sample dataset [13]. Moreover, it has been employed to explore the cognitive profiles of individuals with parkinsonism [14]. For this reason, AP represents a valuable tool for evaluating subjects with ET and normal cognitive functioning, in whom different stages along a continuum of cognitive vulnerability could be hidden.

In the present preliminary, cross-sectional study, we aimed to test the hypothesis that different cognitive profiles may emerge among ET subjects diagnosed as cognitively normal. By applying an innovative data-driven clustering approach, we investigated whether these individuals exhibit subtle performance weaknesses in specific domains that, while still within age- and education-adjusted normative ranges, may reveal latent heterogeneity in cognitive functioning. We also sought to evaluate whether the cognitive subtypes eventually identified within the ET group differ from healthy controls profiles.

## Materials and methods

### Subjects

Fifty subjects, 26 with ET and 24 healthy controls (HC), matched for age, sex and education, were recruited to participate in this study. All participants were consecutively recruited at Neuroscience Research Center of the University “Magna Graecia” of Catanzaro. ET were clinically diagnosticated according to the established criteria [15] and further confirmed based on the integrity of nigrostriatal dopaminergic terminals on dopamine transporter ^123^I-FP CIT-single-photon emission computed tomography (DAT-SPECT) scan [16].

HC were included based on the absence of current neurological or other general medical conditions and no family history of neurological disease.

A careful clinical interview and medical examination was carried out in all subjects to collect demographical and clinical variables; tremor assessment was made according to the Fahn-Tolosa-Marin tremor rating scale (FTM-Scale) [17]. None of the included participants referred subjective cognitive complaints or requests for cognitive evaluation. Cognitive performance was assessed in all participants, and using a comprehensive neuropsychological battery, designed to evaluate multiple cognitive domains, as previously reported [18]: global cognitive functioning was screened using the Mini-Mental State Examination (MMSE) [19]; Frontal and executive functions through the Frontal Assessment Battery (FAB) [20] and Modified Card Sorting Test, considering both Categories Achieved (MCST-CA) and Perseverative Errors (MCST-PE) [21]; Working memory through the Digit Span both Forward (attention span) and Backward (working memory manipulation) [22]; Verbal short- and long- term memory through the Rey Auditory-Verbal Learning Test, Immediate Recall (RAVLT-I) and Delayed Recall (RAVLT-D) [23]; Visuospatial abilities through the Judgments of Line Orientation test – Form V (JLO-V) [24]; Verbal fluency and language comprehension through the Controlled Oral Word Association Test (COWAT) [23] and Token Test [25]. All subjects enrolled in the study scored within normal limits across all tests, based on the Italian normative data and standardized cut-offs for each neuropsychological test [26], taking into account age and education. Moreover, to rule out the presence of significant mood or anxiety disturbances that might act as confounding variables affecting cognitive performance, all subjects were evaluated using Beck Depression Inventory II (BDI-II) [27] and the Hamilton Anxiety Rating Scale (HAMA) [28].

None of the subjects abused drugs or alcohol; furthermore, none of them took medications that could influence cognitive functions; in particular, the subjects with ET did not take benzodiazepines, barbiturates or other drugs whose effect could interfere with cognitive performance.

All participants gave written informed consent to participate at the study, which was approved by the Institutional Ethical Committee of the University “Magna Graecia” of Catanzaro, according to the Helsinki Declaration.

### Statistical analysis

All statistical analyses were conducted using Jamovi (version 2.6.2). To evaluate differences in demographic and cognitive and neuropsychological variables between the ET and HC groups, we performed a one-way ANOVA, while Chi-square test was used for categorical variables (sex distribution). To account for multiple comparisons across the fifteen numerical variables, Bonferroni correction was applied, adjusting the significance threshold accordingly (α = 0.05/15 = 0.0033). Only results meeting this corrected threshold were considered statistically significant.

### Clustering with Affinity Propagation algorithm

To identify latent cognitive subgroups within the ET cohort, we applied AP clustering, an unsupervised machine learning algorithm that autonomously determines the number of clusters based on the internal structure of the data [11]. This method is particularly suited to neurocognitive research, where subtle phenotypic variability may be present even among individuals classified as cognitively normal. Clustering was performed exclusively within the ET group, using raw scores from eleven cognitive variables: MMSE, COWAT, MCST-CA and MCST-PE, RAVLT-I and RAVLT-D, Digit Span Forward, Digit Span Backward, FAB, Token Test, and JLO-V.

The squared Euclidean distance was used as the similarity metric to promote compact and distinct cluster formation. The preference parameter, which influences the number of clusters by determining the likelihood that each data point is selected as an exemplar, was set to the minimum similarity value, following the methodological recommendation [11]. The damping factor was set to 0.5 to ensure convergence and avoid oscillations [29]. While AP is a heuristic and does not guarantee a globally optimal solution, it offers a flexible and efficient approach for detecting naturally occurring subgroups, and it has been successfully applied in prior studies on neurodegenerative and movement disorders [14].

As an additional analysis, we applied AP clustering to the HC group to assess the internal homogeneity of their cognitive profiles. This analysis used the same set of variables, the same similarity metric, and identical parameter settings. All clustering procedures were implemented in Python (v. 3.8.6) using the scikit-learn library (v. 0.23).

Once the clusters within the ET group were identified, we conducted a between-group comparison to examine how each ET subgroup related to the cognitive performance of HC. Specifically, we performed a series of one-way ANOVAs on the cognitive variables across groups (C1, C2, and HC). For each variable showing a significant main effect, we conducted Tukey’s Honest Significant Difference (HSD) tests for post-hoc pairwise comparisons.

This procedure allowed us to determine whether the data-driven cognitive subtypes within the ET group differed meaningfully from healthy individuals, and to identify potential subclinical vulnerability patterns that may not be detectable using conventional diagnostic criteria alone.

## Results

### Group Characteristics

Table 1 summarizes the demographic, clinical, and neuropsychological characteristics of the HC and ET groups. No significant differences were observed between the two groups in terms of age (p = 0.547), sex distribution (female/male: 13/11 in HC vs. 9/17 in ET; p = 0.16, Chi-square test), or education (p = 0.571). No significant group differences were found in global cognition (MMSE), language, visuospatial abilities, executive functions, mood, or anxiety scores (all p > 0.05).

**Table 1:** Demographic, clinical, and neuropsychological characteristics of Healthy Controls (HC) and Essential Tremor (ET) patients. Data are reported as mean ± standard deviation for continuous variables and as counts for categorical variables.


	HC (24)	ET (26)	P-VALUE

**Age**	63.8 ± 6.87	65.3 ± 10.2	0.547^a^

**Sex (F/M)**	13/11	9/17	0.16^b^

**Education**	10.9 ± 4.44	10.2 ± 3.98	0.571^a^

**Familiarity (no/yes)**	N.A.	13/9	N.A.

**Disease_duration**	N.A.	12.7 ± 16.6	N.A.

**FTM-Scale**	N.A.	9.85 ± 4.18	N.A.

MMSE*	27.6 ± 2.08 [24.7–30]	27.5 ± 1.34 [25.7–30]	0.83^a^

COWAT*	29.2 ± 7.61 [17.8–47.6]	26.8 ± 5.84 [17.5–41.5]	0.223^a^

MCST- CA*	5.79 ± 0.721 [3.0–6.0]	5.37 ± 1.3 [3.0–8.7]	0.162^a^

MCST- PE*	0.875 ± 1.61 [0.0–6.4]	1.32 ± 2.04 [0.0–6.4]	0.392^a^

RAVLT_I*	43.5 ± 5.84 [29.7–56.1]	40.8 ± 9.13 [28.9–57.3]	0.217^a^

RAVLT_D*	8.85 ± 2.31 [5.2–15.0]	7.55 ± 2.25 [4.7–12.8]	0.05^a^

Digit_span_F*	5.77 ± 0.875 [4.0–7.2]	5.06 ± 0.719 [3.5–6.25]	**0.003** ^a^

Digit_span_B*	3.79 ± 0.588 [3,4,5]	3.35 ± 0.797 [2,3,4,5]	0.029^a^

FAB*	16 ± 1.46 [13.5–18.0]	15.1 ± 1.74 [13.5–18.0]	0.054^a^

TOKEN*	31.4 ± 1.37 [29.25–33.75]	30.9 ± 2.16 [26.25–33.75]	0.388^a^

JLO-V*	24.5 ± 3.78 [20,21,22,23,24,25,26,27,28,29,30]	23.8 ± 4.43 [20,21,22,23,24,25,26,27,28,29,30]	0.578^a^

**BECK_II**	6.39 ± 4.57	7.19 ± 3.89	0.547^a^

**HAMA**	6.39 ± 3.83	6.85 ± 2.87	0.571^a^


^a^ ANOVA. ^b^ Chi square. In bold significant at Bonferroni’s correction 0.0033. N.A. Not Applicable. Abbreviations: ET = Essential Tremor; HC = Healthy controls; FTM-Scale: Fahn-Tolosa-Marin tremor scale; BDI-II = Beck Depression Inventory II; HAMA = Hamilton Anxiety Rating Scale. Neuropsychological test with possible score range [min-max]: MMSE = Mini Mental State Examination [0–30]; COWAT: Controlled Oral Word Association Test [0 – (no maximus limit)]; MCST-CA = Modified Card Sorting Test-Categories Achieved [0–6]; MCST-PE = Modified Card Sorting Test-Perseverative Errors [0 – (no maximus limit)]; RAVLT-I = Rey Auditory-Verbal Learning Test Immediate Recall [0–75]; RAVLT-D = Rey Auditory-Verbal Learning Test-Delayed Recall [0–15]; Digit_span_F = Digit Span Forward [0–9]; Digit_span_B = Digit Span Backward [0–8]; FAB = Frontal Assessment Battery [0–18]; TOKEN = Token Test [0–36]; JLO-V = Judgment of Line Orientation [0–30]. *Values are reported as mean ± standard deviation [range of observed score as min-max].

A statistically significant difference, surviving to Bonferroni correction for multiple comparisons (adjusted α = 0.0033), emerged only for Digit Span Forward (p = 0.003), where ET patients performing significantly worse than HC. A trend toward lower performance in the ET group was also observed for Digit Span Backward (p = 0.029), RAVLT Delayed Recall (p = 0.050), and the Frontal Assessment Battery (p = 0.054), although these did not reach corrected significance.

### Clustering analysis in healthy controls

As a preliminary step, AP clustering was applied to the HC group to verify the internal homogeneity of their cognitive profiles. The algorithm identified a single cluster, suggesting that healthy individuals formed a uniform group with no evidence of latent cognitive subtypes.

### Clustering analysis in ET

When we applied the AP clustering to the ET group, we identified two distinct subgroups: Cluster 1 (C1, n = 14), characterized by high cognitive performance across all domains, and Cluster 2 (C2, n = 12), which showed selectively impaired scores most prominently evident in memory-related measures (RAVLT-IR, RAVLT-DR), along with a relative weakness in immediate auditory attention (Digit-Span Forward) (Table 2).

**Table 2:** Demographic, clinical, and neuropsychological characteristics of the two ET clusters and healthy controls (HC). Data are reported as mean ± standard deviation for continuous variables and as counts for categorical variables.


	CLUSTER 1 ET (14)	CLUSTER 2 ET (12)	HC (24)	P-VALUE	POST-HOC

**Age**	64.8 ± 9.99	65.8 ± 10.8	63.8 ± 6.87	0.823^a^	N.A.

**Sex (F/M)**	6/8	3/9	13/11	0.25^c^	N.A.

**Education**	10 ± 3.92	10.4 ± 4.21	10.9 ± 4.44	0.823^a^	N.A.

**Familiarity (no/yes)**	5/6	8/3	N.A.	0.19^d^	N.A.

**Disease_duration**	15.2 ± 19.4	10.1 ± 4.97	N.A.	0.408^b^	N.A.

**FTM-Scale**	9.83 ± 12.9	9.50 ± 3.21	N.A.	0.695^b^	N.A.

MMSE*	27.6 ± 1.51 [25.7–30.0]	27.4 ± 1.17 [26.0–30.0]	27.6 ± 2.08 [24.7–30.0]	0.887^a^	N.A.

COWAT*	27.1 ± 7.14 [18.5–41.5]	26.5 ± 4.14 [17.5–32.4]	29.2 ± 7.61 [17.8–47.6]	0.393^a^	N.A.

MCST- CA*	5.41 ± 1.6 [3.0–8.7]	5.33 ± 0.888 [4.0–6.0]	5.79 ± 0.721 [3.0–6.0]	0.281^a^	N.A.

MCST- PE*	1.7 ± 2.4 [0.0–6.4]	0.883 ± 1.52 [0.0–3.7]	0.875 ± 1.61 [0.0–6.4]	0.513^a^	N.A.

RAVLT_I*	48.1 ± 5.38 [40.9–57.3]	32.3 ± 2.68 [28.9–37.6]	43.5 ± 5.84 [29.7–56.1]	**<.0001** ^a^	**C1 > C2** **C1 > HC** **HC > C2**

RAVLT_D*	8.82 ± 2 [6.2–12.8]	6.06 ± 1.51 [4.7–8.6]	8.85 ± 2.31 [5.2–15.0]	**0.0002** ^a^	**C1 > C2** **C1 = HC** **HC > C2**

Digit_span_F*	5.14 ± 0.719 [4.0–6.25]	4.96 ± 0.737 [3.5–6.0]	5.77 ± 0.875 [4.0–7.2]	**0.015** ^a^	**C1 = C2** **C1 = HC** **HC > C2**

Digit_span_B*	3.43 ± 0.938 [2,3,4,5]	3.25 ± 0.622 [2,3,4]	3.79 ± 0.588 [3,4,5]	0.054^a^	N.A.

FAB*	15 ± 2.02 [13.5–18.0]	15.2 ± 1.44 [13.5–17.30]	16 ± 1.46 [13.5–18.0]	0.175^a^	N.A.

TOKEN*	30.4 ± 2.18 [26.25–33.0]	31.6 ± 2.02 [28.0–33.75]	31.4 ± 1.37 [29.25–33.75]	0.272^a^	N.A.

JLO*	22.8 ± 5.45 [20,21,22,23,24,25,26,27,28,29,30]	25.1 ± 2.26 [22,23,24,25,26,27,28,29]	24.5 ± 3.78 [20,21,22,23,24,25,26,27,28,29,30]	0.372^a^	N.A.

**BECK_II**	6.57 ± 3.99	7.92 ± 3.8	6.39 ± 4.57	0.554^a^	N.A.

**HAMA**	6.71 ± 3.31	7 ± 2.37	6.39 ± 3.83	0.85^a^	N.A.


^a^ ANOVA across three groups. ^b^ ANOVA between clusters. ^c^ Chi square across three groups. ^d^ Chi square between clusters. N.A. Not Applicable. In bold results significant at the post hoc. Abbreviations: ET = Essential Tremor; HC = Healthy controls; FTM-Scale: Fahn-Tolosa-Marin tremor scale; BDI-II = Beck Depression Inventory II; HAMA = Hamilton Anxiety Rating Scale. Neuropsychological test with possible score range [min-max]: MMSE = Mini Mental State Examination [0–30]; COWAT: Controlled Oral Word Association Test [0 – (no maximus limit)]; MCST-CA = Modified Card Sorting Test-Categories Achieved [0–6]; MCST-PE = Modified Card Sorting Test-Perseverative Errors [0 – (no maximus limit)]; RAVLT-I = Rey Auditory-Verbal Learning Test Immediate Recall [0–75]; RAVLT-D = Rey Auditory-Verbal Learning Test-Delayed Recall [0–15]; Digit_span_F = Digit Span Forward [0–9]; Digit_span_B = Digit Span Backward [0–8]; FAB = Frontal Assessment Battery [0–18]; TOKEN = Token Test [0–36]; JLO-V = Judgment of Line Orientation [0–30]. *Values are reported as mean ± standard deviation [range of observed score as min-max].

### Comparison between clusters and healthy controls

**Figure 1:**
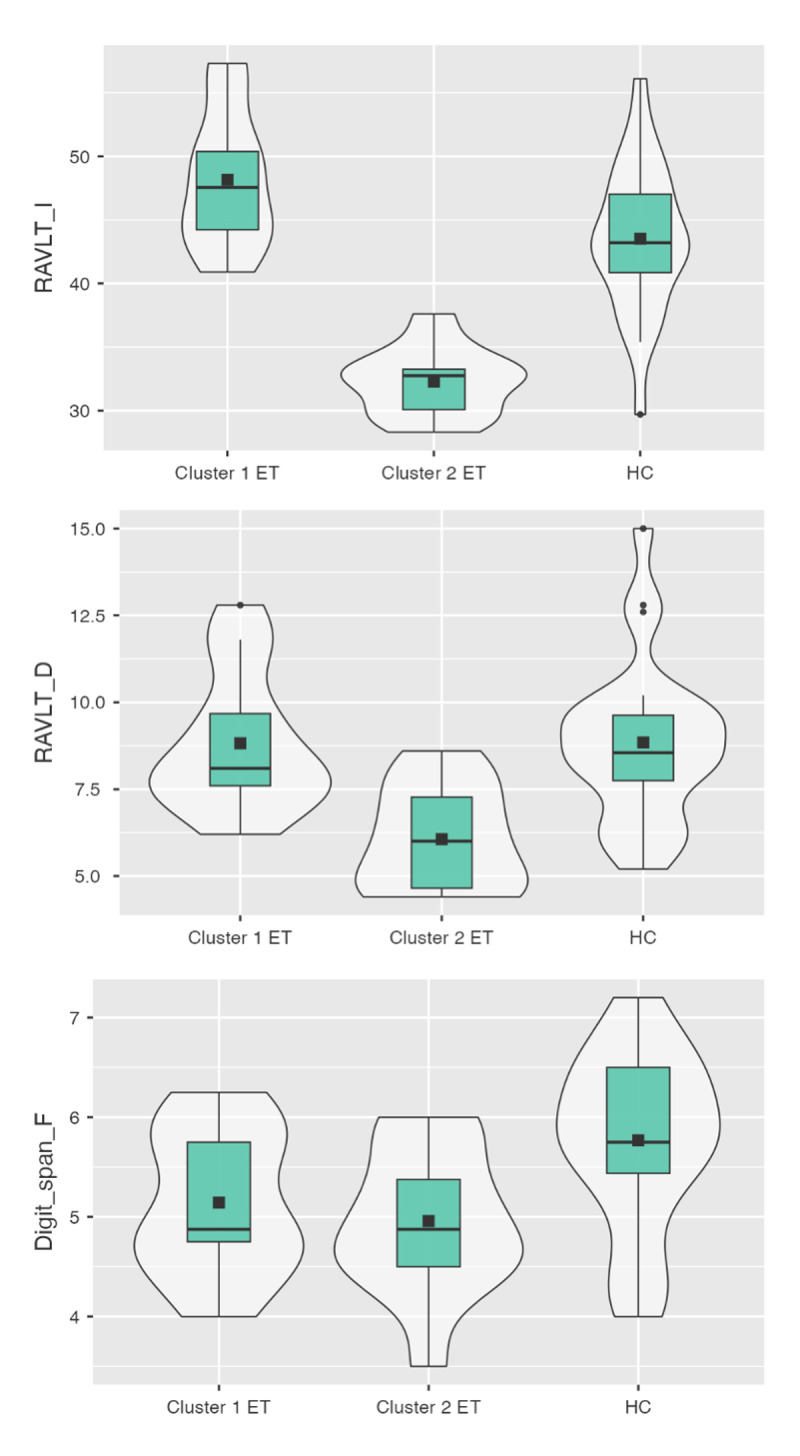
**Violin plots**. Violin plots showing the distribution of scores for the three cognitive variables that showed statistically significant differences in post-hoc comparisons between groups: RAVLT Immediate Recall (RAVLT_I), RAVLT Delayed Recall (RAVLT_D), and Digit Span Forward. The plots compare performance across ET Cluster 1 (C1), ET Cluster 2 (C2), and healthy controls (HC).

A one-way ANOVA comparing C1, C2, and HC (n = 24) revealed significant differences in RAVLT-IR (p < 0.0001), RAVLT-DR (p = 0.0002), and Digit Span Forward (p = 0.015). Tukey post-hoc tests confirmed that C2 performed significantly worse than both C1 and HC on all three measures (p < 0.05, Tukey HSD). No differences were observed between C1 and HC, indicating that individuals in Cluster 1 had a cognitive profile comparable to healthy subjects. The distributions of RAVLT-IR, RAVLT-DR, and Digit Span Forward scores in the C1, C2, and HC groups are shown in Figure 1. A trend toward significance was also observed for Digit Span Backward (p = 0.054), although significance did not survive post-hoc comparisons.

No significant differences emerged across the three groups in other cognitive domains (e.g., executive functions, language, visuospatial abilities), nor in affective symptoms (BDI-II, HAMA).

Regarding demographic and clinical characteristics, we found no significant differences in age (p = 0.823), education (p = 0.823), or sex distribution (p = 0.25, Chi-square across groups) across the three groups. Tremor severity, disease duration, and familiarity for ET did not differ between C1 and C2 ET clusters. Detailed results are reported in Table 2.

## Discussion

This study aimed to explore cognitive heterogeneity in a cohort of cognitively normal individuals ET patients by applying a data-driven clustering approach, to investigate potential cognitive profiles that are not captured by conventional neuropsychological classification and to evaluate how ET subgroups relate to age-matched HC. Using Affinity Propagation clustering we discovered the existence of two distinct cognitive subgroups within ET, mainly distinguished by memory performance, opposite to what we observed in the HC group, which was a cognitively homogeneous group, forming a single cluster. The ET cluster characterized by lower scores in memory tests, also had a relative weakness in immediate attention with performance significantly different not only from other ET subjects, but also from the control group, suggesting a profile of potential cognitive vulnerability.

Through the unsupervised clustering approach, we were able to capture two main profiles within our ET population: the C1 groups, including 14 patients, was characterized by high performance across all domains, while the C2, comprising 12 patients, showed a compromised profile in scores evaluating verbal memory and auditory recall domains. Importantly, all ET participants enrolled in this study were classified as cognitively normal based on clinical criteria and standard neuropsychological thresholds. None met criteria for mild cognitive impairment (MCI) or dementia [30]. But, despite being within the normal ranges, C2 displayed significantly worse performance on both immediate and delayed verbal memory tasks (RAVLT-IR and -DR), as well as in auditory short-term memory and immediate attention, as measured by Digit Span Forward.

This evidence mirrors the findings of a previous clustering study in which the authors also found memory to be the most affected domain in one of clusters of ET patients they identified [10]. In agreement with previous results [10,31], our patients in C1 and C2 were not different in disease duration and tremor scores, thus suggesting that these clinical features may not be primary determinants of cognitive status. Regarding the age and education level, which are well-known modifiers of cognitive reserve and risk factors for cognitive decline in ET population [32,33], we did not find significant differences between C1 and C2, nor in terms of sex distribution. Overall, these findings indicate that the observed cognitive differences are unlikely to be explained by demographic or clinical confounds and instead may reflect a difference intrinsically linked to the cognitive functioning of the subjects examined.

Further in agreement with the cognitive profile previously described [10], in addition to verbal memory deficits, our patients in C2 cluster exhibited a significant reduction in Digit Span Forward performance compared to both C1 and HC. This finding highlights that the cognitive vulnerability observed in this subgroup of ET patients extends beyond verbal memory, affecting also immediate auditory attention. Indeed, Digit Span Forward is typically considered a measure of attention span and short-term auditory memory, because it requires retention and reproduction of verbal information without manipulation [34]. In contrast, differences in Digit Span Backward, which better reflects working memory, showed only a trend toward significance and did not survive post-hoc comparisons. This pattern reinforces the interpretation that the primary weakness in C2 lies in attentional and encoding processes, rather than in higher-order executive functions.

The key novelty of this study is represented by the inclusion of a control group, which allowed us to contextualize the clinical relevance of cognitive different profiles observed in ET. Notably, C2 subjects not only performed worse than their C1 counterparts, but also displayed significantly lower scores compared to HC, primarily in verbal memory tasks, alongside an involvement of attentional processes, indicating a deviation from normal reference values. On the contrary, ET subjects in the high cognitive functioning cluster, had scores perfectly corresponding to those of HC. Furthermore, when we performed the cluster analysis, based on the analogous cognitive variables, in the HC group, we found a single cluster, highlighting that the same heterogeneity observed in ET was not present in healthy subjects of comparable age, sex and education. All these findings suggest that the differences in cognitive performance may be interpreted as the identification of a potential risk phenotype within the ET population, rather than just variability.

On the other hand, the lack of heterogeneity within the HC group may appear somewhat unexpected, given that a proportion of the general aging population will also develop MCI and, in some cases, dementia, albeit at a lower rate than is typically observed in ET [8]. Several factors may account for this finding. One possibility is a different timeframe of risk expression: cognitive vulnerabilities may manifest earlier in ET due to disease-related mechanisms, while similar changes in healthy individuals might emerge later in the aging process. Another potential explanation involves differences in etiology, with ET-specific alterations, such as cerebello-thalamo-cortical dysfunction, leading to earlier or more selective cognitive deficits. Finally, the relatively small sample size may have limited the detection of low-prevalence cognitive subgroups.

Overall, the results of our study are particularly relevant also in the debate still open about the risk for MCI and dementia development in ET. Regarding the specific cognitive affected domains, deficits in executive function and attention are the most frequently reported by large number of studies [35], often attributed to the involvement of the cerebello-thalamo-cortical circuit [31,36,37]. Several of the same studies, also highlighted the presence of memory impairments in ET, even in patients without overt dementia [35,38]. These deficits often involve verbal memory and delayed recall, suggesting possible dysfunction in medial temporal lobe structures, like hippocampus, and networks beyond cerebellar pathways. Neuroimaging studies have supported this view, showing structural and functional changes spreading within these regions [16,39,40,41]. Additionally, large-scale case-control studies and meta-analyses have reported significantly worse performance on memory-related subtests of cognitive screening tools (e.g., MoCA and MMSE) in ET patients compared to healthy individuals [42], thus raising the hypothesis that in a subset of patients, ET might coexist with, or even represent, a prodromal phase of neurodegenerative conditions typically associated with memory impairment, such as Alzheimer’s disease [43]. Supporting this possibility, data from a large, population-based cohort showed that ET patients with episodic memory impairment, assessed via a simple word recall task, had an increased risk of mortality (HR = 1.25) compared to both HC and ET patients without memory deficits. Although no significant additive interaction was found between ET and memory impairment, these findings suggest that episodic memory deficits may serve as an independent marker of increased vulnerability and may modestly amplify mortality risk in individuals with ET [44]. Taken together, these data reinforce the notion that memory dysfunction in ET is not an incidental finding, but it may reflect underlying pathological mechanisms toward the degenerative continuum.

Our findings also have significant translational value. Indeed, the possibility to identify a cognitive risk profile among individuals who are otherwise deemed “normal” by standard neuropsychological screening has significant implications in the clinical environment. Our results highlight consistent and domain-specific cognitive vulnerabilities, particularly in memory, that may represent a potential marker of a prodromal stage of cognitive decline or indicate a distinct ET subtype with increased risk for neurodegeneration. This could impact the capability to stratify patients early, through simple routine cognitive tests, identifying subjects eligible for closer cognitive follow-up, preventive strategies, and rehabilitation programs aimed at preserving cognitive function.

The main limitation of the present study is its cross-sectional design, which prevents us from drawing conclusions on the longitudinal trajectories of the identified clusters. Although we cannot establish whether the subgroup of ET patients with relatively poorer memory and attention performance will progress to MCI or dementia, the inclusion of a matched HC group strengthens the validity of our findings by showing that such heterogeneity was specific to ET and not observed in HC. Future longitudinal studies will be crucial to assess the prognostic value of the identified cognitive profiles. Moreover, the generalizability of our findings is limited due to the relatively small sample, thus the proportion of ET patients classified in the memory-impaired cluster reflects only the distribution within our selected cognitively normal sample and therefore cannot be generalized to the broader ET population.

However, our work has methodological strengths in the rigorous patient enrollment, in terms of clinical evaluation with scintigraphic confirmation and neuropsychological assessment (normal cognition across all tests, no medications affecting cognition), and statistical approach. Indeed, the clustering analysis model we used does not require predefining the number of clusters, allowing the structure to emerge directly from the data, enhancing the interpretability of results when the underlying structure of the data is unknown.

Future studies will extend our findings. One perspective of our work could be integrating neuroimaging and biological markers to clarify the neuroanatomical substrates underpinning these cognitive differences and to determine whether the low memory performance profile corresponds to specific structural or functional brain changes. In addition, the inclusion of genetic factors such as APOE genotype and Alzheimer’s disease–related blood biomarkers would be valuable to explore whether this ET subgroup shares biological vulnerability pathways with prodromal Alzheimer’s disease.

In conclusion, our findings add evidence that subtle cognitive changes could occur in ET with normal cognition, with memory dysfunction emerging as a key discriminative feature, along with a relative immediate attention weakness. The discovery of a homogeneous profile in the control group, opposite to the cognitive stratification observed within ET, and the differences observed between one of the ET clusters and the other groups suggest the existence of distinct cognitive phenotypes in ET, that may not simply reflect normal variability but indicate a cognitively vulnerable phenotype, potentially subclinical, within the spectrum of ET.
